# Occupational Accidents Caused by Sharp Objects Among Healthcare Workers and Medical Students: A Retrospective Observational Study

**DOI:** 10.7759/cureus.110228

**Published:** 2026-06-04

**Authors:** Christina Gkirtsou, Evangelia Nena, Eftychia Kontekaki, Theocharis Konstantinidis, Gregory Trypsianis, Periklis Panagopoulos, Theodoros Konstantinidis

**Affiliations:** 1 School of Medicine, Blood Transfusion Center, University General Hospital of Alexandroupolis, Alexandroupolis, GRC; 2 Laboratory of Social Medicine, Department of Medicine, Democritus University of Thrace, University General Hospital of Alexandroupolis, Alexandoupolis, GRC; 3 Miscellaneous, Blood Transfusion Center, University General Hospital of Alexandroupolis, Alexandroupolis, GRC; 4 University Laboratory of Microbiology, Democritus University of Thrace, University General Hospital of Alexandroupolis, Alexandroupolis, GRC; 5 Laboratory of Medical Statistics, Department of Medicine, Democritus University of Thrace, Alexandroupolis, GRC; 6 2nd University Department of Internal Medicine, and Department of Infectious Diseases, HIV Unit, Democritus University of Thrace, University General Hospital of Alexandroupolis, Alexandroupolis, GRC; 7 Laboratory of Hygiene and Environmental Protection, Department of Medicine, Democritus University of Thrace, University General Hospital of Alexandroupolis, Alexandroupolis, GRC

**Keywords:** healthcare workers (hcws), medical students, needlestick injuries, occupational exposure, sharp object injuries (sois)

## Abstract

Objectives: This study aimed to retrospectively evaluate sharp object injuries (SOIs) among healthcare workers (HCWs) and medical students in a 10-year period, from 2014 to 2023. A secondary aim was to assess the procedures and the prevalence of exposure to bloodborne pathogens.

Methods: The data collection was retrieved from the hospital records for the examined period. All laboratory parameters had been determined by the electrochemiluminescence method and were performed using the Alinity instrument (Abbott Diagnostics, IL, USA). Statistical analysis was performed with IBM SPSS Statistics version 30.0.

Results: In total, 771 occupational accidents occurred, involving individuals with an average age of 34 years. Blood sampling was the most common procedure (n=360; 46.7%). Regarding the source, 61 (7.9%) cases involved abandoned sharps, while in 710 (92.1%) cases the source patient was identified. Laboratory analysis of the identified source patients showed that 31 (4.4%) were HBsAg-positive, 25 (3.5%) were anti-HCV positive only, 4 (0.6%) were HIV I/II positive, and 8 (1.1%) had screening tests for *Treponema pallidum* that were positive. Of note, two (0.3%) HCWs and two (0.9%) medical students were found to have pre-existing anti-hepatitis C antibodies at baseline testing; however, no true seroconversion occurred after exposure.

Conclusions: This retrospective observational study underscores the persistent clinical risk of SOIs among HCWs and medical students.

## Introduction

Healthcare workers (HCWs) are the cornerstone of a resilient healthcare system, and their primary goal is to improve the health of patients. However, they face significant occupational hazards worldwide [1], including sharp object injuries (SOIs), which can expose workers to more than 20 different blood-borne pathogens, resulting in over one thousand infections each year worldwide, the most common of which are hepatitis B and C, human immunodeficiency virus (HIV), and *Treponema pallidum* [2-5]. These incidents not only jeopardize the physical and psychological well-being of the medical staff but also impose a substantial financial burden on healthcare systems due to post-exposure prophylaxis, laboratory testing, and potential long-term treatment [3,4].

In 2022, through analysis of WHA74.14, the World Health Organization (WHO) recognized the need for substantial protection and reinforcement of the occupational health and safety of HCWs, acknowledging that a safe and protected workforce is of paramount importance for a comprehensive and effective healthcare system, contributing to the minimization of patient harm. National policy, in accordance with the Global Patient Safety Action Plan 2021-2030, adopted by the 74th World Health Assembly, should be committed to establishing objectives related to the protection of the health and safety of HCWs [6,7].

​Despite the implementation of universal precautions and the introduction of safety-engineered devices, the incidence of SOIs remains high. This is particularly relevant in high-pressure environments such as internal medicine departments, which often account for a significant proportion of reported incidents due to the high volume of invasive procedures and the complexity of patient care. Furthermore, the vast majority of studies indicate that anxiety, depression, high levels of burnout, and the improper handling of sharps among HCWs are significantly correlated with an increased risk of injuries, as well as compromised standards of patient care and safety [5,6].

Moreover, medical doctors frequently emerge as the professional group most affected by these injuries, highlighting the critical need for targeted safety protocols and specialized training within both internal medicine and surgical departments, as well as emergency units. The literature suggests that underreporting is a major obstacle in accurately assessing the epidemiological profile of these injuries, with rates of unreported incidents varying significantly across different clinical settings [8,9]. Therefore, long-term retrospective analysis of reported incidents is vital for identifying high-risk groups, evaluating the effectiveness of existing safety protocols, and implementing targeted educational interventions.

​The aim of this study was to record and investigate the characteristics of occupational injuries from SOIs among HCWs and sixth-year medical students at a 700-bed tertiary care university hospital in Northeastern Greece, as well as to identify the relevant risk factors.

## Materials and methods

This retrospective study was conducted at the University General Hospital of Alexandroupolis, a major academic referral center in the Balkan region, analysing data from January 2014 through December 2023.

Data were obtained from the healthcare personnel (i.e., physicians, nurses, medical laboratory technicians, health visitors, physiotherapists, cleaning and sterilization staff, as well as 6th-year medical students) who had a history of a needlestick or other sharp object injury during that period and who had consented to the use of their data for scientific purposes.

The exclusion criteria were the following: occupational injuries stemming from falls, electrocution, or other non-sharp objects (excluding needles or other sharp instruments), or injuries reported by individuals who were not classified as HCWs.

Prior to conducting any data analysis or reporting, confidentiality was ensured, and all personal identifiers, such as names, addresses, phone numbers, and all potentially characteristic information of the HCWs, were removed.

Statistical analysis

The collected data were subjected to a comprehensive statistical analysis using the IBM SPSS Statistics version 30.0. Descriptive statistics were applied to summarize the study population’s characteristics. Specifically, frequencies (N) and percentages (%) were calculated to determine the prevalence of needlestick injuries (NSIs), normalized both by the annual number of reported accidents and the total number of HCWs employed at the facility.

The incidence of injuries was stratified and analysed per calendar year, gender, age group, professional specialty, and clinical department. Furthermore, the analysis extended to the circumstances of the injury and the immediate post-exposure actions taken by the personnel. Regarding the immunological status of the participants, the prevalence of vaccination coverage and the presence of protective antibody titers were evaluated.

In terms of clinical risk assessment, the serological status of the source patients was analysed for bloodborne pathogens, including HIV, HBV, HCV, and *Treponema pallidum*. Finally, the frequency of post-exposure prophylaxis (PEP) administration among HCWs was calculated to assess the clinical management of high-risk exposures. For all inferential statistical tests, a P-value of <0.05 was considered statistically significant.

## Results

This retrospective observational study analysed 771 records of HCWs employed in the university hospital, who had been injured with a sharp object during the period 2014-2023. Women accounted for 69.9% of SOIs (p<0.001), primarily among staff under 40 (74.2%). Most incidents occurred during blood sampling (46.7%) within internal medicine departments (49.9%), with physicians and students most affected.

Prevalence of accidents

A total of 771 incidents were recorded during the study period. The highest annual prevalence rates of SOIs occurred in 2016 (93 out of 771, 12.1%) and 2022 (94 out of 771, 12.2%), while the lowest was observed in 2018 (37 out of 771, 4.8%) and 2017 (58 out of 771, 7.5%) (Table 1). The χ2 test demonstrated that the reduction in prevalence during the 2017-2018 biennium compared to the other years was statistically significant (χ2 = 36.717, df = 9, p < 0.0001). 

**Table 1:** Annual prevalence of sharp object injuries among HCW (2014-2023) Note: N, number; (Accident rate = number of accidents /total HCWs) x 100 HCW: healthcare workers

Years	Accidents; N (%)	HCWs (N)	Accident rate among all employees (%)
2014	76 (9.9%)	1205	6.3
2015	81 (10.5%)	1206	6.7
2016	93 (12.1%)	1214	7.7
2017	58 (7.5%)	1218	4.8
2018	37 (4.8%)	1237	3.0
2019	82 (10.6%)	1209	6.8
2020	74 (9.6%)	1255	5.9
2021	92 (11.9%)	1248	7.4
2022	94 (12.2%)	1242	7.6
2023	84 (10.9%)	1277	6.6

**Figure 1:**
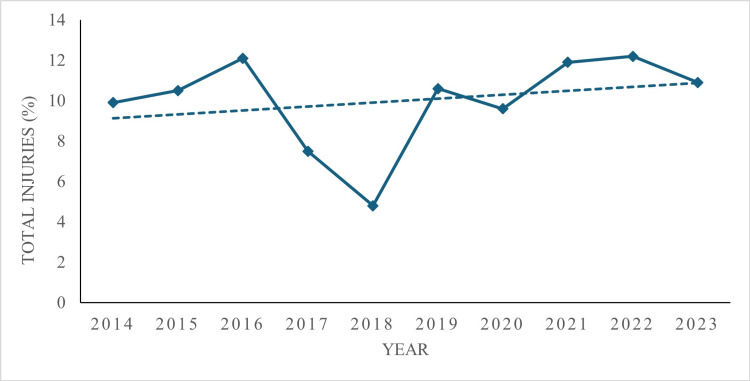
Annual distribution of sharp object injuries as a percentage of total recorded accidents (N=771) While the overall 10-year trend shows no statistically significant increase or decrease (r=0.260, p=0.469) the specific reduction observed during 2017-2018 was statistically significant (χ2=36.717, df=9, p<0.0001)

Nevertheless, no statistically significant upward or downward trend was observed over time, either in the number of accidents (correlation coefficient r = 0.260, p = 0.469) or in the prevalence rate (correlation coefficient r = 0.255, p = 0.477) as illustrated in Figure 1.

**Figure 2:**
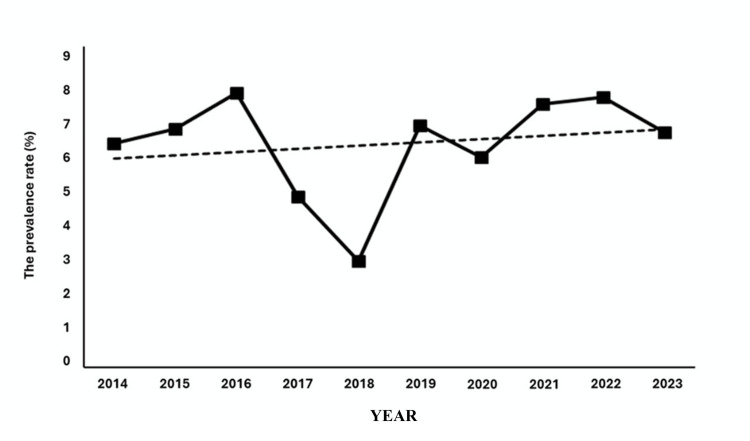
The prevalence rate of injuries caused by sharp objects per year among all employees The solid line represents the annual prevalence rate of injuries, while the dashed line indicates the overall trend over the 10-year study period (2014-2023) Abbreviations: %, percentage

The longitudinal analysis of the annual prevalence rates among all HCW revealed certain fluctuations over the 10-year period. Although a statistically significant decrease was noted in 2018, the overall trend demonstrated a slight upward inclination throughout the study duration. These annual variations and the corresponding trend line are depicted in Figure 2.

Prevalence of accidents in relation to gender

**Figure 3:**
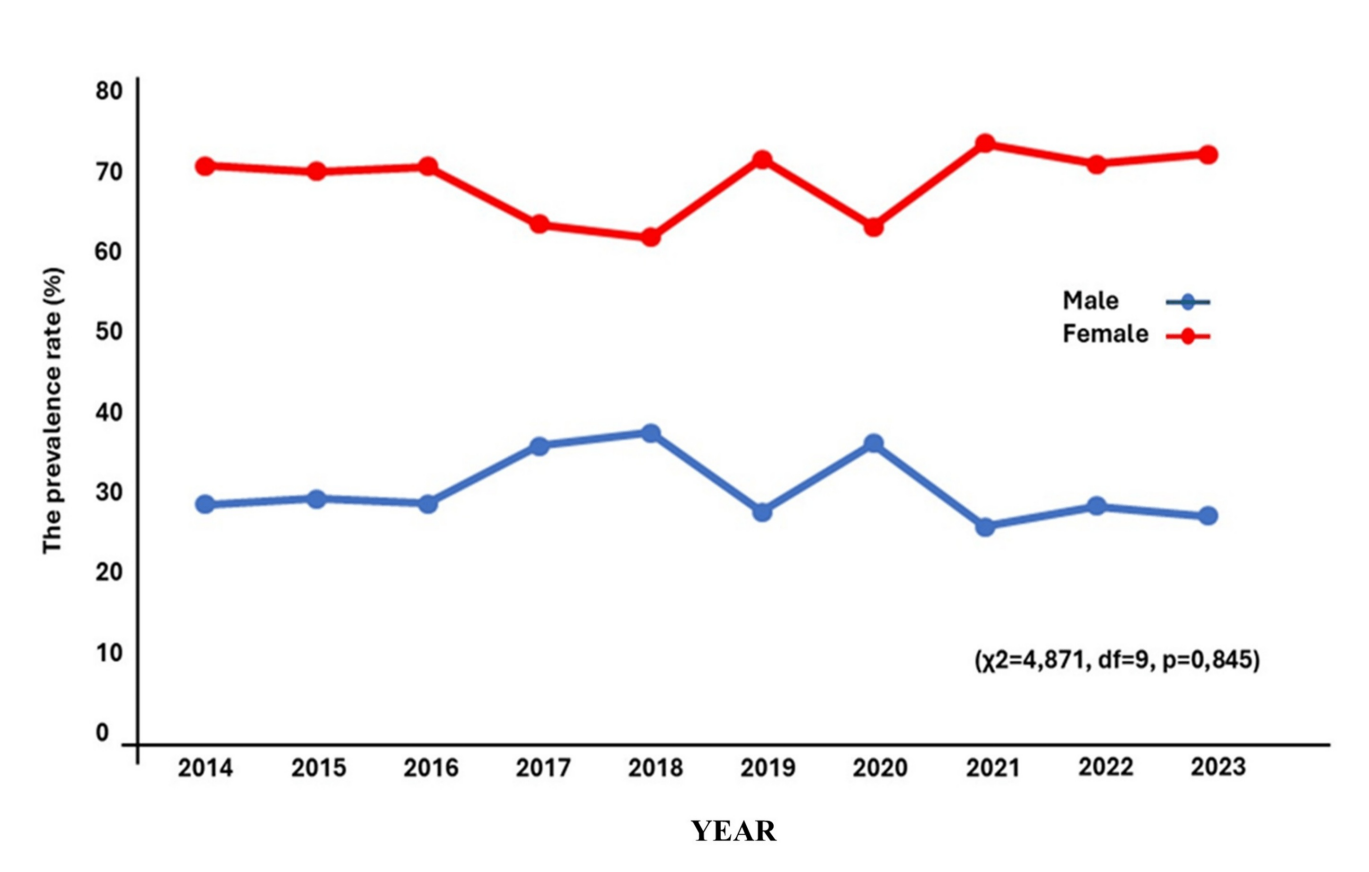
Distribution of occupational accidents caused by sharp objects in relation to gender in all monitoring years Note: The graph illustrates the annual distribution of sharp object injuries by gender. Statistical analysis shows no statistically significant difference in the distribution pattern between males and females over the study period. Abbreviations: χ2, chi-squared test; df, degrees of freedom; p, probability value.

Of the 771 incident reports, 69.9% involved women (539 reports) and 30.1% involved men (232 reports). The χ2 test showed that SOIs were statistically significantly more common in women than in men (χ2=122.242, df=1, p<0.0001). The highest prevalence of accidents in women was observed in 2021 (68 out of 92 reports, 73.9%), while in men it was recorded in 2018 (14 out of 37 reports, 37.8%). As shown in Table 2, both the detailed annual distribution and the graphical representation of these trends (Figure 3) indicate that the distribution of incidents by gender remained relatively stable throughout the study period (χ2=4.871, df=9, p=0.845).

**Table 2:** Annual distribution of sharp object injuries by gender (2014-2023) Note: N, number of accidents; percentages represent the proportion of injuries within each year by gender. Abbreviations: Pearson’s chi-squared test (χ2=4.871, df=9, p=0.845).

Year	Males, N (%)	Females, N (%)	p-value
2014	22 (28.9%)	54 (71.1%)	-
2015	24 (29.6%)	57 (70.4%)	-
2016	27 (29.0%)	66 (71.0%)	-
2017	21 (36.2%)	37 (63.8%)	-
2018	14 (37.8%)	23 (62.2%)	-
2019	23 (28.0%)	59 (72.0%)	-
2020	27 (36.5%)	47 (63.5%)	-
2021	24 (26.1%)	68 (73.9%)	-
2022	27 (28.7%)	67 (71.3%)	-
2023	23 (27.4%)	61 (72.6%)	-
Total	232 (30.1%)	539 (69.9%)	0.845

Prevalence of accidents in relation to age

**Figure 4:**
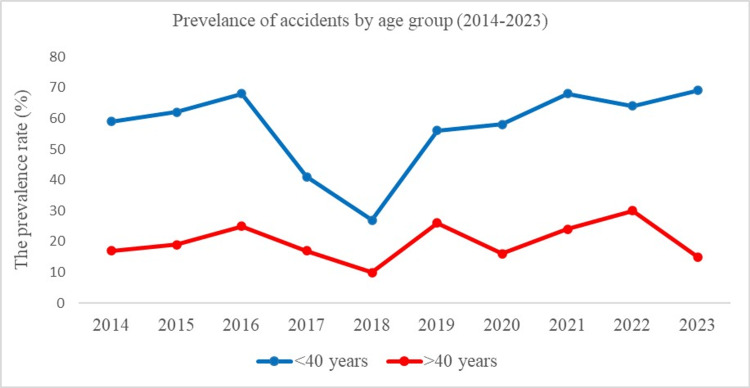
Prevalence of occupational accidents caused by sharp objects in relation to age in a university hospital in Northeastern Greece across all years of follow-up Note: The graph illustrates the annual prevalence rate of sharp object injuries stratified into two age groups: younger (≤ 40 years) and older (> 40 years) employees. The incidence was consistently higher in the age group (<40 years) throughout the study period. No statistically significant change in the age distribution was observed over 10-year period (p=0.360) Abbreviations: %, percentage.

Statistical analysis demonstrated that the prevalence of sharp object injuries decreased significantly as age increased (χ2=294.499, df=3, p<0.0001). HCWs aged 40 years or younger accounted for most incidents (572 out of 771, 74.2%), while those over 40 years old accounted for a significantly lower proportion (199 out of 771, 25.8%). A notable trend was observed among younger HCWs, whose accident rates remained consistently high throughout the study period. Detailed annual data by age group and corresponding longitudinal trends are illustrated in Table 3 and Figure 4. Despite annual variations, the overall distribution of incidents by age group remained consistent across the full study duration (χ2=29.016, df=27, p=0.360).

**Table 3:** Distribution of occupational accidents caused by sharp objects in relation to age (Total 2014-2023) Note: Data represent the total number of occupational incidents and their respective percentages across different age groups. Statistical significance for the annual distribution was assessed using Pearson's chi-squared test (χ2=29.016, df=27, p=0.360).

Age Group	Total Incidents (n=771)	Percentage (%)
≤30 years	385	49.9
31-40 years	187	24.3
41-50 years	134	17.4
>50 years	65	8.4
Total	771	100
p-value		0.360

Prevalence of accidents by profession

Regarding the distribution of injuries by category, the highest prevalence rates throughout the study period were consistently observed among final-year medical students. The χ2 test showed that sharp object injuries were statistically significantly more common among doctors, nurses, and medical students compared to the other occupations (χ2=657.973, df=5, p<0.0001). Specifically, the prevalence rate for medical students ranged from 18% (2018) to a peak of 39% (2023). In contrast, much lower rates were recorded for physicians (2.7% to 9.9%) and nurses (1.6% to 5.8%). To substantiate the use of prevalence rates, complete annual denominator data, representing the total number of active individuals within each professional category, were analyzed for each year of the 10-year study period (2014-2023) in Table 4.

**Table 4:** Annual prevalence of sharp object injuries among Physicians, Nursing and Medical Students (2014-2023) Note: n, number of accidents; N, total number of HCWs/students per year; Accident rate= (n/N) x 100

Years	Physicians n/N (%)	Nursing n/N (%)	Medical Students n/N (%)
2014	29/400 (7.3%)	19/531 (3.6%)	19/71 (27%)
2015	35/392 (8.9%)	22/525 (4.2%)	19/84 (23%)
2016	38/383 (9.9%)	25/526 (4.8%)	22/102 (21.6%)
2017	26/381 (6.8%)	12/560 (2.1%)	18/77 (23.4%)
2018	10/368 (2.7%)	9/566 (1.6%)	16/89 (18%)
2019	25/364 (6.9%)	29/549 (5.3%)	24/71 (34%)
2020	36/375 (9.6%)	16/567 (2.8%)	16/84 (19%)
2021	37/381 (9.7%)	32/555 (5.8%)	22/86 (25.6%)
2022	40/405 (9.9%)	29/559 (5.1%)	22/71 (31%)
2023	28/415 (6.7%)	24/562 (4.2%)	30/77 (39%)

**Figure 5:**
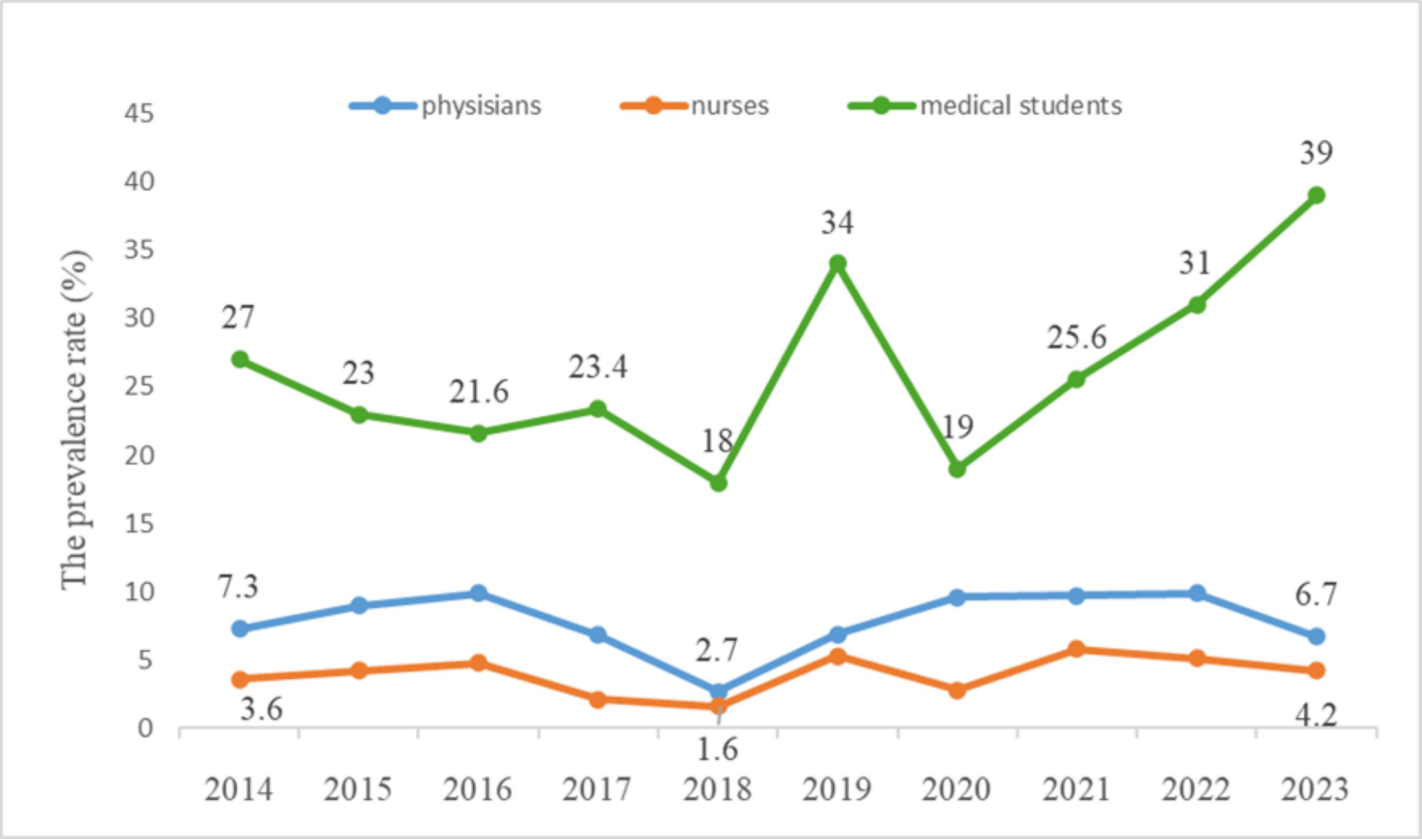
Annual prevalence of sharp object injuries by professional category (2014-2023) Note: The graph illustrates the annual prevalence rate of sharp object injuries stratified by professional category (physicians, nurses and medical students). Medical students consistently demonstrated the highest prevalence rates compared to other healthcare groups. No statistically significant longitudinal trend was observed for any category (physicians: p=0.781; nurses: p=0.410; medical students: p=0.132. Abbreviations: %, percentage.

Detailed annual prevalence rates by professional category and corresponding longitudinal trends are illustrated in Figure 5. No statistically significant upward trend was observed over time for any of the main professional categories (physicians: p= 0.781; nurses p=0.410; medical students: p=0.132.

Prevalence of accidents in relation to the incident segment

Regarding the department where the incident occurred, it was shown that this was most common in internal medicine clinics (385 out of 771, 49.9%), followed by surgical clinics (258 out of 771, 33.5%), intersectoral departments and support units (98 out of 771, 12.7%) and the laboratories (17 out of 771, 2.2%) (Table 5). Notably, out of the 771 documented cases of injuries, 641 (83%) were caused by hollow-bore needles.

**Table 5:** Distribution of occupational accidents caused by sharp objects by department *Intersectoral departments: emergency department (ED), short-stay unit (SSU). **Other departments: Accidents involving improperly discarded needles found in general waste bins, hospital corridors, or soiled linen. Note: Statistical significance was determined using Pearson's chi-squared test (χ2=687.178, df=4, p<0.0001).

Departments	Number of injuries N (%)	p-value
Internal medicine	385 (49.9%)	-
Surgery	258 (33.5%)	-
Intersectoral departments*	98 (12.7%)	-
Laboratory	17 (2.2%)	-
Other departments**	13 (1.7%)	-
		<0.0001

The χ2 test showed that sharp object injury was statistically significantly more frequent in the internal medicine pathological and surgical clinics compared to the other departments (χ2=687.178, df=4, p<0.0001).

The χ2 analysis was employed to detect statistically significant differences in the frequencies of incidents among the various subgroups, rather than to establish causal correlations, revealing a clear differentiation between the employees’ occupation and the department where the accidents occurred (χ2=24.962, df=8, p=0.002). More specifically, although the largest number of incidents occurred in internal medicine clinics for both physicians (149 out of 304, 49.0%) and nurses (97 out of 217, 44.7%) and medical students (124 out of 208, 59.6%), occupational accidents caused by sharp objects in internal medicine clinics were more frequent among students than among physicians and nurses. Conversely, occupational accidents caused by sharp objects in intersectoral departments and support units were more frequent among physicians (40 out of 304, 13.2%) and nurses (39 out of 217, 18.0%) than among medical students (13 out of 208, 6.3%).

Prevalence of accidents in relation to the (diagnostic/ therapeutic) procedure

The most common procedure leading to a sharp object injury (SOI) was blood draw or arterial blood gases collection (360 out of 771, 47%), followed by surgery with a suture needle or scalpel (104 out of 771, 13.5%). In a very small percentage, the cause of injury was not reported (2 out of 771, 0.3%) (Table 6).

**Table 6:** Distribution of occupational accidents caused by sharp objects in relation to the procedures that led to injury Note: Statistical significance was assessed using Pearson's chi-squared test (χ2=961.555, df=7, p<0.0001)

Procedure	Number of injuries N (%)	p-value
Injury with a suture needle or scalpel during surgery	104 (13.5%)	<0.0001
Injury with a needle during blood sampling or blood gas collection	360 (46.7%)	-
Injury with a needle after blood sampling-	127 (16.5%)	-
Injury with a needle while recapping	75 (9.7%)	-
Injury with a needle while discarding it in the yellow box	16 (2.1%)	-
Injury with a needle or scalpel that penetrated the yellow box from an unknown patient	24 (3.1%)	-
Uncovered needle found on the floor, on the sheets, in a yellow or black garbage bag from an unknown patient	63 (8.2%)	-
Not reported	2 (0.3%)	-

The χ2 test showed that SOIs were statistically significantly more common during blood collection or blood gas collection compared to the other procedures (χ2=961.555, df=7, p<0.0001).

Significant differences in SOI prevalence were also observed across professions and procedures (χ2=115.278, df=14, p<0.001). Blood or gas collection was the primary cause of injury for physicians, 46.4% (141/304), nurses, 52.5%(114/217), and medical students, 49.5%(103/208). While surgical injuries from needles or scalpels were more frequent among physicians (13.2%), medical students showed higher rates of injuries during blood collection (21.6%) and recapping (20.7%). Notably, cleaning workers reported the highest injury rate (94.4%), mainly due to improperly discarded needles found on floors, in liners, or in garbage bags. Regarding post-exposure management, 44.5% (343/771) of the injured HCWs and medical students did not have their subsequent post-exposure measures documented in the institutional database. Among those who acted, the most frequent measure was washing the site with plenty of water and antiseptic or alcohol (n=162), while only six individuals took no action. Detailed data on the measures taken by healthcare professionals and medical students are presented in Table 7 (343 out of 771). 

**Table 7:** Measures taken by healthcare professionals and final year medical students after being injured by sharp objects at a university hospital in Northeastern Greece Note: Data represents the measures and actions taken by healthcare workers and medical students immediately following an occupational injury caused by sharp objects. Abbreviations: N, frequency

Measures taken after injury	Number of cases (n)
Not reported	343
Disinfection with alcohol and povidone iodine	40
Disinfection with betadine, antiseptic and bleach	57
Pressure on the site and immersion in bleach	57
Washing with plenty of water and antiseptic or alcohol	162
Pressure on the area and washing with soap and water	49
Washing with povidone iodine or immersion	57
No action taken	6
Total	771

Immunological status

Regarding the immunological status of healthcare personnel (400 out of 563, 71%), the majority of employees reported being vaccinated against hepatitis B, as were the final-year medical students (159 out of 208, 76%). A very small number of medical students and HCWs had not been fully vaccinated against hepatitis B (8 out of 208, 4%) and (28 out of 563, 5%), respectively. Of the 28 HCWs who were not vaccinated, 5 had positive antibody titers against hepatitis B. Of the 208 medical students, 41 (20%) reported that they did not remember or did not know if they had been vaccinated, however, 35 (85%) of them had antibodies against hepatitis B. Similarly, 135 out of 563 (24%) health workers reported that they did not remember or did not know if they had been vaccinated against hepatitis B, however, of these, 102 (75.5%) had developed antibodies against hepatitis B (Table 8).

**Table 8:** Hepatitis B vaccination and immune status of healthcare workers (n=563) and medical students (n=208) following sharp object injuries (2014-2023)

Vaccination status (reported)		Number of injuries N (%)
Health workers (n=563)		
“I have been vaccinated”	Total	400 (71%)
	Antibody positive	369
	Antibody negative	31
“I don't know / I don't remember”	Total	135 (24%)
	Antibody positive	102
	Antibody negative	33
“I have not been vaccinated”	Total	28 (5%)
	Antibody positive	5
	Antibody negative	23
Medical students (n=208)		
“I have been vaccinated”	Total	159 (76%)
	Antibody positive	139
	Antibody negative	20
“I don't know / I don't remember”	Total	41 (20%)
	Antibody Positive	35
	Antibody Negative	6
“I have not been vaccinated”	Total	8 (4%)
	Antibody Positive	0
	Antibody Negative	8

**Figure 6:**
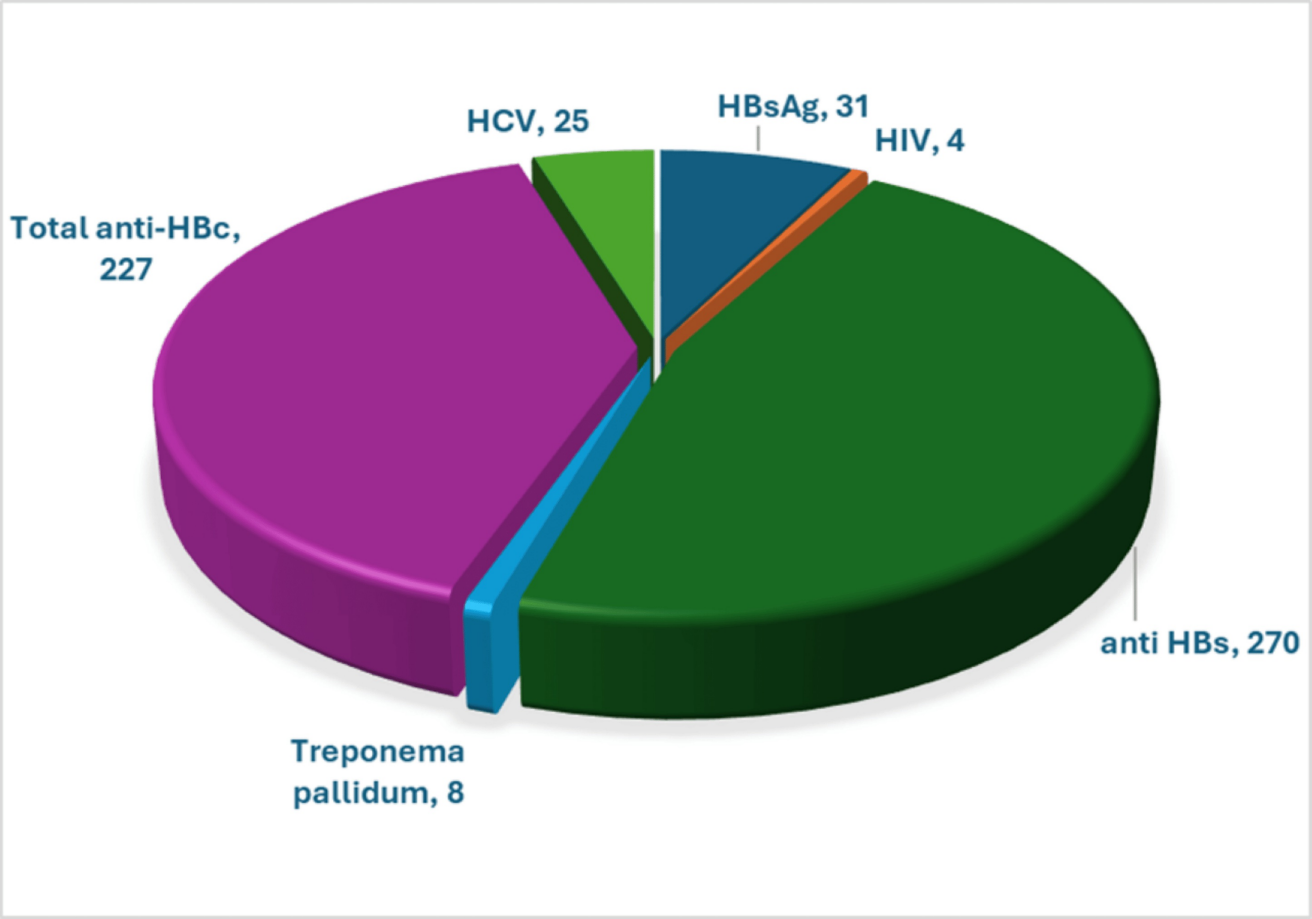
Data of virological tests of source patients in a tertiary hospital (2014-2023) Note: The pie chart displays the frequency of positive virological and serological markers identified in source patients during the 10-year follow-up period. "HBc total" refers to total anti-HBc antibodies (IgG and IgM combined), which serve as a serological marker of exposure to the hepatitis B virus.

Of the 771 cases of source patients, 61 (7.9%) were associated with needles from unknown sources, found in bed linens, garbage bags, on the floor, or protruding from the disposal box. Regarding virological testing of the remaining 710 source patients, 31 (4.4%) were positive, and 679 (95.6%) were negative for the hepatitis B surface antigen (HBsAg), of which 144 (20.3%) had natural immunity. The number of source patients who were immune after vaccination numbered 126 (18%), while 25 (3.5%) had an acute infection. Of the 25 patients who tested positive for HCV antibodies, all had received treatment without risk of transmitting the disease. Four source patients tested positive for human immunodeficiency virus (HIV); consequently, an HCW and a medical student required post-exposure prophylaxis (PEP), which was initiated with ISENTRESS (400mg) and a combination of TDF/FTC (tenofovir/emtricitabine) for one month. Additionally, *Treponema pallidum* was identified in 8 patients. The distribution of virological and serological markers among source patients is summarized in Figure 6.

Regarding hepatitis B, the overall immunity rate was 38% (270/710) among the source patients, whereas high immunity rates were observed among HCWs, 84.5% (476/563), and medical students, 83.7% (174/208). Screening of source patients identified a baseline prevalence of hepatitis C virus (HCV) at 3.5%, Treponema pallidum at 1.1%, and HIV at 0.6%. The inclusion of Treponema pallidum serology was performed in accordance with the established institutional post-exposure management protocol, which mandates comprehensive baseline screening of source patients to ensure maximum occupational safety. Crucially, no occupational seroconversion for HCV or HIV occurred among the exposed healthcare personnel or medical students during the study period. Regarding the immunological status of the HCWs and medical students, the positive anti-HCV cases detected during the screening represented pre-existing, baseline infections rather than new transmissions. Follow-up monitoring confirmed the absence of any occupational seroconversion among the exposed healthcare staff and medical students. These findings, which are detailed in Table 8, underscore the pathogen load in source patients and highlight the imperative need for strict post-exposure protocols.

**Table 9:** Immune status of healthcare workers, medical students and patients with HBV, HCV, and HIV in a University hospital in Greece

Immune status	Patients	HCWs	Medical students
HBV immunity	270	476	174
HBsAg positive only	31	0	0
Anti-HCV positive	25	2	2
Anti-HIV positive	4	0	0
HBsAg and anti-HCV positive	1	0	0
Treponema pallidum	8	0	0

## Discussion

According to official data of the Human Resources Department of the University Hospital, the nursing staff represents the largest share of the workforce, and their job description includes procedures with a high probability of injury, such as venous catheter placement or intramuscular and subcutaneous injections, etc. However, the descriptive data presented in our study indicate that physicians (39.4%) among health care workers and sixth-year medical students between January 2014 and December 2023 were the group that exhibited the highest number of reported injuries caused by sharp objects. This is followed by the nurses (28.1%) and medical students (27%), while the lowest injury frequencies were recorded among scientific staff (0.1%). This distribution is consistent with several observational studies, which note high reporting numbers among medical personnel [8,9].

Out of the 771 documented cases of injuries, 641 (83%) were caused by hollow-bore needles, followed by suture needles, 104 (13.5%). Most of the recorded incidents occurred during either blood sampling or arterial blood gases sampling (n=360; 46.7%). These procedures are primarily performed by physicians in this setting, and the result is fully consistent with the above-mentioned percentage, which indicates that physicians were the group most affected. This is highly relevant as hollow-bore needles, which are commonly used for blood collection, are associated with an increased risk of HIV transmission in the event of injury from a seropositive source patient, as argued in the study by Stamataki et al. and Cardo et al. [10,11].

The findings of this study, regarding the types of incidents with the highest number of reported injuries caused by sharp objects, show internal medicine clinics with a rate of up to 50% (385). This percentage indicates that the risk of exposure depends on the intensity of the work and the frequency of the procedures performed by specific occupational groups. This aligns with findings from a study by Cardo et al., which also associates occupational exposure risk with workload intensity and the frequency of invasive procedures [10]. Other studies show that nurses have a high rate of injuries, perhaps because physicians have been found to be less likely to report their injuries compared to other healthcare staff [9,12,13].

It is noteworthy that final (sixth)-year medical students, while having a lower injury rate (27%) compared to doctors and nurses, are at higher risk of injury. These injuries, although they may not be attributed to occupational burnout, as medical students are occupied for 47 weeks of their clinical training (389 hours), while an average HCW works 48 weeks (1920 hours), are most likely due to factors such as limited clinical experience, developing manual skills, and a lack of knowledge about infection prevention and control [14].

The results of this study are consistent with the study by Elisa et al., which showed a high prevalence of needle stick injuries among medical students and highlighted the need for awareness and education of students regarding infection control during their clinical training [15].

In this study, the observed distribution of occupational accidents caused by injuries from sharp objects showed a decrease with older age. This pattern aligns with the observation that younger HCWs may often have less clinical experience to navigate potential hazards in their workplace. The speed and intensity with which they perform their tasks can lead to carelessness and distraction. In addition, employees with limited clinical experience may more frequently perform high-volume or unfamiliar, dangerous tasks, which reflects the higher frequency of reported injuries in these subgroups. These descriptive trends are consistent with previous surveillance studies by Medeni and Nyberg et al [16,17].

In our study, despite the high injury rates, there was no seroconversion in any of the HCWs who encountered stray needles or patients infected with HBV, HCV, HIV, or *Treponema pallidum*. This may be due to immediate reporting and intervention after the incident with the administration of appropriate treatment regimens, which, according to studies, are tolerated by the body (e.g., TDF/FTC Truvada Tabl Lop/rit Kaletra sir) or P6P TDF/FTC, Tb ISENTRESS 400mg), significantly reducing the risk of HIV transmission [18]. This finding is in line with Directive 2010/32 of the Council of the European Union of May 10, 2010, and is confirmed by various studies which report that the risk of transmission of blood-borne pathogens is significantly reduced when standard precautions are applied directly by HCWs [5,9,19].

In the cases analysed, of the 563 employees, 400 (71%) stated that they had been vaccinated against hepatitis B; however, according to the results of the virological test, 476 (84.5%) were immune to HBV. Similarly, of the 208 sixth-year medical students, 159 (76%) stated that they had been vaccinated against hepatitis B, while the results of the virological test showed that 174 (83.7%) were immune to HBV. Our hospital's vaccination program and the recommendations of the infection control committee are contributing to the increased immunization rate among healthcare personnel.

In the context of occupational accidents in the healthcare sector, our retrospective study reveals a compelling yet perplexing trend: the subsequent utilization of bleach by employees. This practice warrants critical examination, as it potentially compounds primary injuries or stems from misguided protocols post-incident. Our findings are consistent with those reported by Stamataki et al., highlighting a significant need for improved and systematic training on occupational safety, alongside the establishment of clearer, more comprehensive guidelines to effectively reduce the occurrence of such injuries in clinical practice in the future [11,20].

The findings of this retrospective observational study highlight a consistent longitudinal trend in utilization patterns post-incident within the healthcare sector. These observations underscore the importance of continuous adherence to established occupational safety protocols and the integration of comprehensive tracking systems. Our data support the utility of aligning institutional practices with international safety standards, such as those outlined by WHO [6]. Ensuring the systematic availability of safety-engineered devices, alongside structured clinical training, remains a relevant component of institutional health surveillance frameworks aimed at monitoring healthcare-associated exposures [21,22].

Study limitations

Despite the significance of the findings, there are limitations in this study that must be acknowledged. Primarily, the reliance on officially reported incidents may result in an underestimation of the true incidence of needlestick injuries, as under-reporting remains a widespread phenomenon among HCWs due to factors such as perceived lack of time or the underestimation of infection risks.

Furthermore, due to the unavailability of annual institutional denominator data stratified by biological sex, we were unable to calculate sex-specific prevalence rates for occupational injuries. Consequently, the analysis regarding sex is restricted to absolute frequencies and percentages of reported cases, which should be interpreted with caution as they do not account for the baseline gender distribution of the hospital personnel.

Moreover, the single-center scope of the study also limits the generalizability of the results to other healthcare settings with different safety infrastructures or staffing levels. Finally, the analysis did not account for potential longitudinal variations in safety training protocols or the introduction of new medical devices that may have occurred during the decade-long study interval.

## Conclusions

This 10-year retrospective observational analysis underscores that monitoring sharp object injuries is essential for supporting healthcare safety within institutional frameworks. The descriptive findings provide a detailed epidemiological mapping across medical specialties, age groups, and training levels, highlighting distinct frequency patterns that can guide targeted educational interventions. Aligning institutional practices with international safety guidelines and ensuring the systematic availability of safety-engineered devices remain critical parameters. Maintaining a comprehensive registry framework is vital for evaluating reporting trends, ensuring precise documentation, and fostering occupational safety and systemic resilience within the university hospital setting. Such an approach is crucial to mitigate the high risk identified throughout our investigation. By prioritizing institutional protocols and safety goals, healthcare facilities can ensure a safer environment that fosters both professional well-being and systemic resilience. 
